# Combining Genomic and Phenomic Information for Predicting Grain Protein Content and Grain Yield in Spring Wheat

**DOI:** 10.3389/fpls.2021.613300

**Published:** 2021-02-12

**Authors:** Karansher S. Sandhu, Paul D. Mihalyov, Megan J. Lewien, Michael O. Pumphrey, Arron H. Carter

**Affiliations:** ^1^Department of Crop and Soil Sciences, Washington State University, Pullman, WA, United States; ^2^Dewey Scientific, Pullman, WA, United States; ^3^United States Forest Service, Eugene, OR, United States

**Keywords:** genomic selection, grain protein content, high throughput phenotyping, multivariate models, nested association mapping, secondary traits, wheat

## Abstract

Genomics and high throughput phenomics have the potential to revolutionize the field of wheat (*Triticum aestivum* L.) breeding. Genomic selection (GS) has been used for predicting various quantitative traits in wheat, especially grain yield. However, there are few GS studies for grain protein content (GPC), which is a crucial quality determinant. Incorporation of secondary correlated traits in GS models has been demonstrated to improve accuracy. The objectives of this research were to compare performance of single and multi-trait GS models for predicting GPC and grain yield in wheat and to identify optimal growth stages for collecting secondary traits. We used 650 recombinant inbred lines from a spring wheat nested association mapping (NAM) population. The population was phenotyped over 3 years (2014–2016), and spectral information was collected at heading and grain filling stages. The ability to predict GPC and grain yield was assessed using secondary traits, univariate, covariate, and multivariate GS models for within and across cycle predictions. Our results indicate that GS accuracy increased by an average of 12% for GPC and 20% for grain yield by including secondary traits in the models. Spectral information collected at heading was superior for predicting GPC, whereas grain yield was more accurately predicted during the grain filling stage. Green normalized difference vegetation index had the largest effect on the prediction of GPC either used individually or with multiple indices in the GS models. An increased prediction ability for GPC and grain yield with the inclusion of secondary traits demonstrates the potential to improve the genetic gain per unit time and cost in wheat breeding.

## Introduction

Agronomically important traits are often controlled by a large number of small effect quantitative trait locus (QTLs), which have been challenging to take advantage of in plant breeding (Bernardo, 2008). In recent years, genome-wide association studies (GWAS) have offered a solution for dissecting the genetic basis of complex traits like disease resistance, grain yield, and end-use quality traits (Jernigan et al., 2018; Lewien et al., 2018). However, in GWAS, small effect QTLs are challenging to map and even if the mapping is successful, their effect is usually confounded due to multiple QTLs present. Moreover, the small effect of these QTLs makes them inefficient to be used with marker-assisted selection (MAS) (Bernardo, 2008). Grain yield is an essential example of a quantitative trait which is difficult to improve in nearly all crop plants. On that regard, genomic selection (GS) has demonstrated the capacity to overcome the limitation of MAS and quantitative traits and is being implemented in various crop plants to improve genetic gain through selection (Jannink et al., 2010).

Originally proposed by Meuwissen et al. (2001), GS provides an alternative method for predicting the breeding values in plants using genome-wide markers. It offers the potential for accelerating genetic gain by increasing selection intensity, accuracy, and shortening the breeding cycle time. To perform GS, a population that has been both genotyped and phenotyped is selected and termed as the training population. Training populations are used to train the GS models for estimating marker effects, which are then used to assign genomic estimated breeding values (GEBVs) for lines which have not been phenotyped. This second set of lines which have only been genotyped are termed as the testing population (Crossa et al., 2010; Rutkoski et al., 2014). A model’s ability to predict accurately is termed as prediction accuracy and is defined as the correlation between observed phenotypes and predicted breeding values. Individuals can be selected based on the GEBVs before being tested under field conditions, ultimately speeding up the breeding cycle (Heffner et al., 2010; Burgueño et al., 2012). Numerous factors affect GS accuracy, including sample size, heritability, selection intensity, relatedness between training and testing population, and genotype imputation methods (Heffner et al., 2011; Isidro et al., 2015).

Genomic selection models rely on accurate phenotypic information which has been the main driver for increasing genetic gain in classical breeding approaches. Prediction accuracy of GS models depends upon the quality of phenotypic data collected on the training population (Beyene et al., 2019). However, advancements in phenotyping has lagged compared with recent advancements in genomics (White et al., 2012). During the last decade, several high throughput phenotyping (HTP) tools have been developed to cope with the phenotyping bottleneck (White and Conley, 2013; Araus and Cairns, 2014). Recently, HTP tools have been implemented to measure various traits in wheat breeding programs, such as vegetation indices, growth rate, plant height, and disease resistance (Stewart et al., 2016; Jimenez-Berni et al., 2018; Khan et al., 2018; Rincent et al., 2018). HTP could be performed during different growth stages and in multiple environmental conditions, drastically increasing phenotypic data to improve selection accuracy (Lopes et al., 2012; Singh et al., 2016). In wheat, secondary traits aid in indirect selection for primary traits such as grain yield, although these estimates might not provide the same accuracy as that of direct selection (Gizaw et al., 2018b,c). These indirect estimates for selection are of great value in early generation breeding cycles when the seed is limited to take measurements for quantitative traits and conduct multi-environment trials. Therefore, prediction of quantitative traits at an early stage using HTP and genome-wide markers may assist in improving selection accuracy.

The basis of spectral radiometry is to measure electromagnetic energy at varying wavelengths interacting with different plant parts. Plant cells, tissues, and metabolites have a light specific reflectance, absorption, and transmittance of photons (Tucker and Sellers, 1986; Sankaran et al., 2010). The phenotype is measured quantitatively through interaction between light and plants, that may aid in differentiating healthy and stressed plants. Spectral reflectance indices (SRI) are derived by measuring photons in visible and near-infrared regions of the electromagnetic spectrum. These indices provide information about different physiological and agronomic traits of plants. The SRI calculated from reflected light in these regions can be categorized into three main groups. In the first group, a combination of reflection form visible and near-infrared region is used to derive SRIs. These indices provide information about stay green duration, vegetative greenness, photosynthetic efficiency, and rate of senescence (Babar et al., 2006). This group contains indices such as simple ratio (SR) and normalized difference vegetation index (NDVI). The second group contain indices such as anthocyanin reflectance index (ARI) and photochemical reflectance index (PRI), which are solely derived from reflectance in the visible region and estimate the abundance and composition of plant pigments (Peñuelas et al., 1994). The indices in the third group are calculated from reflectance in the near-infrared region and provide information about plant hydration status. Most commonly used indices for measuring water stress are water index (WI) and normalized water index (NWI) (Penuelas et al., 1993; Zarate-Valdez et al., 2012).

Combining information from SRI and GS to identify lines which have higher genetic potential for grain yield and end-use quality traits have the potential for use in wheat breeding. There are various physiological processes affecting grain yield and grain protein content (GPC) in wheat, and SRI provides indirect information about them (Gutiérrez-Rodríguez et al., 2004; Babar et al., 2006). Traditionally, most of the GS models were single trait models, including phenotypic information about primary traits only, such as grain yield, GPC, end-use quality attributes, or disease resistance, which are of main interest to the plant breeder. These single trait GS models do not take advantage of the correlation between the primary trait of interest and secondary traits like SRI, which indirectly explain the physiological processes occurring in the plants. Recently, multi-trait GS models have been applied to utilize the power of correlated traits (Anche et al., 2020). Improvement in prediction accuracies for traits having lower heritability has been observed by including correlated traits in the multivariate GS models (Jia and Jannink, 2012). Calus and Veerkamp (2011) showed that multivariate GS models increase the prediction accuracy up to 0.12 using traits having a high genetic correlation. Even traits having less genetic correlation improved the prediction accuracy when incorporated in multi-trait GS models. Crain et al. (2018) showed that incorporation of vegetation indices and canopy temperature in multivariate GS models improve the accuracy for grain yield in wheat by as much as 50% compared to a univariate GS model.

These findings show that incorporation of secondary traits helps improve the performance of GS models. Grain yield and GPC are two traits very important in hard red spring wheat breeding, yet difficult to select for due to their well-known negative correlation. Furthermore, these traits are correlated to various SRI and can be used for indirect selection. Thus, we wanted to evaluate the (1) comparison between univariate and multivariate GS models for predicting GPC and grain yield in wheat; (2) identification of the best growth stage for collecting spectral information in wheat breeding in the Pacific Northwest (PNW) for each trait; (3) selection of the best SRI for incorporation into GS models; and (4) assessment of GS model performance for within and across environments predictions for both traits.

## Materials and Methods

### Plant Material

The nested association mapping (NAM) population used in this study consists of 32 spring wheat accessions from the USDA-ARS National Small Grains Collection, each crossed to common cultivar “Berkut” to create 32 half-sib families (Blake et al., 2019). Berkut was used as a common parent because it is a semi-dwarf and broadly adapted photoperiod insensitive cultivar released by the International Maize and Wheat Improvement Center (CIMMYT), Mexico. As most of the NAM parents were non-adapted landraces, crosses with “Berkut” helps in evaluating effects of various un-adapted alleles. Recombinant inbred lines (RILs) were generated by the single seed descent method for five generations. A total of 864 *F*_2_ plants from each family were planted in greenhouse in Bozeman, MT in 2010. Selections were performed from the *F*_2_ to *F*_5_ generation to select early flowering plants and photoperiod insensitive genotypes. Furthermore, plants having height greater than the median height were discarded, as most of the landraces were homozygous for *Rht* alleles. Complete details about the population development is referred to Blake et al. (2019). At the end, seventy-five early heading and semi-dwarf plants were selected from each cross resulting in a population of 2,400 RILs whose genotyping data were provided by Kansas State University (Jordan et al., 2018). Due to space constraint, 650 RILs from the original 2,400 were planted between 2014 and 2016 at Spillman Agronomy Farm, Pullman, WA, United States (Sandhu et al., 2020). A modified augmented design was used each year with 15–20% of the field plots planted with the replicated checks (Berkut, “McNeal” (Lanning et al., 1994), and “Thatcher”).

### Trait Measurement and Calculations

Grain yield (tha^–1^) was obtained from grain weight per plot with a Wintersteiger Nursery Master combine (Ried im Innkreis, Austria). A Perten DA 7,000 NIR analyzer (Perkin Elmer, Sweden) was used to determine the percentage of GPC. A handheld CROPSCAN multi-spectral radiometer (CROPSCAN, Inc., Rochester, United States) was used to obtain spectral reflectance at Feekes growth stages 10.1 (heading) and 11.1 (grain filling) (Large, 1954). These two stages were used for selecting spectral information, as previous studies from our group showed that these stages have high correlation to primary traits of interest (Gizaw et al., 2016, 2018b). These two stages provide a window of 1 week when spectral data could be collected. We continuously monitored the weather conditions and regularly visited the field to monitor growth stages, in order to decide the best day for data collection. CROPSCAN was radiometric calibrated before utilization, which accounts for any interference due to clouds and wind during the operation. CROPSCAN contains selective filters which measures incident and reflected radiation at 16 different wavelengths between 420 and 980 nm. During data collection, CROPSCAN was placed in the middle of each plot around 1 meter above the canopy level. Data for spectral reflectance was taken within a 2-h window of solar noon and avoiding shadow, clouds, and strong wind. Reflectance values from the whole plots were averaged to obtain the single value for a particular genotype to avoid the bias. Spectral information for each plot was processed through the CROPSACN MSR software. Eight SRI were derived using reflectance values (Table 1).

**TABLE 1:** Spectral reflectance indices, their calculation, and physiological processes measured for a nested association mapping population of spring wheat.

Index	Formula^+^	Physiological processes	References
Normalized difference vegetation index (NDVI)	(R^800^ − R^680^)/(R^800^ + R^680^)	Biomass, plant health, vegetative greenness	Rouse et al., 1972
Normalized water index (NWI)	(R^970^ − R^880^)/(R^970^ + R^880^)	Plant water status	Prasad et al., 2007
Water index (WI)	R^970^/R^900^	Plant water status, root access to moisture	Penuelas et al., 1993
Simple ratio (SR)	R^800^/R^680^	Green biomass, degree of senescence	Stenberg et al., 2004
Green normalized difference vegetation index (GNDVI)	(R^780^ − R^550^)/(R^780^ + R^550^)	Chlorophyll content	Gitelson et al., 1996
Photochemical reflectance index (PRI)	(R^531^ − R^570^)/(R^531^ + R^570^)	Carotenoid content	Peñuelas et al., 1997
Normalized chlorophyll pigment ratio index (NCPI)	(R^680^ − R^430^)/(R^680^ + R^430^)	Chlorophyll pigments	Peñuelas et al., 1994
Anthocyanin reflectance index (ARI)	R^800^ (1/R^550^ − 1/R^700^)	Anthocyanin pigment	Gitelson et al., 1996

*^+^The letter “R” followed by three-digit number stands for reflection measures from that wavelength.*

### Statistical Analysis

The augmented complete block design (ACBD) model implemented in the R program was used to calculate adjusted means for all phenotypic data collected under field conditions between 2014 and 2016 (Rodríguez et al., 2018). Best linear unbiased estimates (BLUE) were calculated for each environment, treating effects as fixed and using the model

$${\text{Y}}_{\text{ij}}=\text{u}+{\text{Block}}_{\text{i}}+{\text{Gen}}_{\text{j}}+{\text{Check}}_{\text{j}}+{\text{e}}_{\text{ij}}.$$

Where Y_ij_ is the primary trait, u is the mean effect, Block_i_ represents effect of the *i*th block, Check_j_ denotes effect of the repeated checks on each block, Gen_j_ represents un-replicated genotypes, and e_ij_ is the standard normal error. Broad sense heritability for all traits is obtained from the ACBD model treating genotypic effects as random and using the formula $H^{2}=\sigma_{\text{g},/}^{2}\,\left(\sigma_{\text{g}}^{2}+\sigma_{\text{e}}^{2}\right)$.

Where *H*^2^ is the broad-sense heritability, σ^2^_*g*_ and σ^2^_*e*_ are the genotypic and error variance components.

Narrow sense heritability for both primary and secondary traits were calculated with the model

Y=Xb+Zg+e

Where Y is the BLUP of the genotype for each trait, X is an incidence matrix for the fixed effect (b), Z is also an incidence matrix corresponding to random genetic effect (g), and e is the standard error. Variance and covariance were based on the assumptions that g ∼ *N*(0, Gσ^2^_*a*_), where G is the genomic relationship matrix and σ^2^_*a*_ is the additive genetic variance, and e ∼ *N*(0, Iσ^2^_*e*_), where I is the identity matrix and σ^2^_*e*_ is the residual variance. Narrow sense heritability for primary and secondary traits was calculated using the formula h^2^ = $\frac{\sigma^{2}\,a}{\sigma^{2}\,a+\sigma^{2}\,e}.$

Genetic correlation (*r*_*g*_) was calculated with the bivariate model, which is represented as

$$\left[\begin{array}{c} y_{A}\\ y_{B} \end{array}\right]=\left[\begin{array}{cc} X_{A} & 0\\ 0 & X_{\text{B}} \end{array}\right]\,\left[\begin{array}{c} b_{A}\\ b_{B} \end{array}\right]+\left[\begin{array}{cc} Z_{A} & 0\\ 0 & Z_{B} \end{array}\right]\,\left[\begin{array}{c} g_{A}\\ g_{B} \end{array}\right]+\left[\begin{array}{c} {}_{\epsilon_{A}}\\ {}_{\epsilon_{B}} \end{array}\right]$$

Where y_*A*_ and y_*B*_ are BLUP for primary and secondary traits, respectively, *X* and *Z* are fixed and random design matrix, subscript A and B represent the primary trait (GPC or grain yield) and secondary trait (one of the SRI), separately, and b, g, and e are the fixed effects, random genetic effect, and residual for each trait, respectively. Variance components were calculated assuming $\left[\begin{array}{c} g_{A}\\ g_{B} \end{array}\right]$∼ *N*(0, H ⊗ G), where H is the genetic variance-covariance matrix and G is genomic relationship matrix, and $\left[\begin{array}{c} \epsilon_{A}\\ \epsilon_{B} \end{array}\right]$∼ *N*(0, I ⊗ R), where I is identify matrix, and R is the residual variance covariance matrix. Genetic correlation was calculated as

$$r_{G}=\frac{c\,o\,v\,\left(A,B\right)}{\sqrt{v\,a\,r\,\left(A\right)\cdot v\,a\,r\,\left(B\right)}}$$

Where *cov*(*A*,*B*) is the covariance between primary and secondary trait, and var(A), and var(B) represents the genetic variance of the primary and secondary trait, individually (SAS Institute Inc, 2011).

### Genotyping

Genotyping by sequencing and 90K iSelect SNP genotyping was used for genotyping the whole NAM population (Poland et al., 2012; Wang et al., 2014). Detailed procedures about genotyping, marker calling, and map construction is reported in Jordan et al. (2018). Initial genotyping data consisted of 73,345 molecular markers anchored to the Chinese Spring RefSeqv1 reference map at Kansas State University (Marcussen et al., 2014; Jordan et al., 2018). Individual lines missing phenotypic data in one environment were removed before genotype filtering. Markers were discarded if more than 20% of the lines had missing data, and lines that had more than 10% genetic data missing were removed for further analysis. Furthermore, markers were discarded with minor allele frequency less than 0.1. At the end of quality filters, 40,005 polymorphic markers were remaining for 635 individuals.

### Genomic Selection Models

Genomic selection emerged as a technique that avoided using an individual marker for predicting a trait, as in the MAS and QTL mapping. Meuwissen et al. (2001) proposed GS to use the whole genome-wide markers for estimating the marker effects and total genetic values, thus minimizing the biasedness during marker effect estimation. However, a large number of markers (p) and fewer individuals (n) created the so-called “large p, small n” problem. Ordinary least squares could not estimate the marker effects due to a lack of enough degrees of freedom. Furthermore, high collinearity among the markers results in the overfitted model. Several statistical models were proposed to overcome these limitations for using whole genome-wide markers, which can be grouped into variable selection models, shrinkage models, dimension reduction methods, and kernel methods. The commonly used variable selection models are Bayes A, Bayes B, Bayes C, shrinkage models are LASSO, rrBLUP, and elastic net. Dimension reduction methods include principal component analysis and partial least square, and finally, kernel methods include reproducing kernel hilbert space and support vector machine. All these GS models can be represented as

$$y_{i}=g\,\left(x_{i}\right)+e_{i}$$

Where *y*_*i*_ is the observed phenotype for a particular trait in *i*th individual, *x*_*i*_ is a vector of 1 × *p* predictors (markers), *g*(*x*_*i*_) is a function relating the predictors to the phenotypes, and *e*_*i*_ is the residual term. Each GS model tries to lower the residuals or certain loss function. The GEBVs estimated from the models are equal to *g*(*x*_*i*_).

The traditional least square regression for predicting a trait and their residual sum of the square is represented as

$$\text{RSS}=\sum_{i=1}^{n}{\left(y_{i}-\beta_{0}-\sum_{j=1}^{p}\beta_{j}\,x_{i\,j}\right)}^{2}$$

Ridge regression is very similar to least square regression, except the coefficients of the equations are estimated using minimizing the ridge regression coefficient estimated as

$$\sum_{i=1}^{n}{\left(y_{i}-\beta_{0}-\sum_{j=1}^{p}\beta_{j}\,x_{i\,j}\right)}^{2}+\lambda \,\sum_{j=1}^{p}\beta_{j}^{2}=\mathrm{RSS}+\lambda \,\sum_{j=1}^{p}\beta_{j}^{2}$$

Where λ is known as the tuning parameter. We can see that ridge regression fit the data by lowering the RSS and selecting small coefficients for $\sum_{j=1}^{p}\beta_{j}^{2}$ to shrink the estimates toward zero, and hence in this way, it uses the shrinkage penalty. The value of λ can vary from zero to infinity and is usually selected by cross-validation.

Another way of solving for *λ* in GS is assuming that marker effects are drawn from a normal distribution centered on zero and solving the mixed linear model equation of Henderson (1975). Here λ = σ^2^_*e*_/σ^2^_*u*_ where σ^2^_*e*_ is the residual variance and σ^2^_*u*_ is marker effect variance, as small σ^2^_*u*_ will result in shrinking of marker effects strongly toward zero, showing that large λ has an equivalent impact too. Here in this study, we used the rrBLUP package for performing GS using shrinkage capacity of the ridge regression; and this model is equivalent to the traditional BLUP models, and hence all the formulas are represented as mixed model equations.

The genome-wide marker effects for GPC and grain yield were estimated using ridge regression best linear unbiased prediction (rrBLUP) including SRI in the model (Endelman, 2011). Predictions were made using the rrBLUP GS model in the R package “rrBLUP,” according to the model:

y=μ+Z⁢u+e

Where y is the N × 1 vector of BLUEs for the phenotypic trait, μ is the overall mean, Z is an N × M matrix linking markers to the genotypes, μ is the vector of normally distributed random marker effects as μ∼ *N*(0, Iσ^2^_*u*_) and e is the residual error with e ∼ *N*(0, Iσ^2^_*e*_). The solution for mixed equation can be written as

$$\text{u}={\text{Z}}^{\text{T}}\,{\left({\text{ZZ}}^{\text{T}}+\lambda \,\text{I}\right)}^{-1}\,\text{y}$$

Here λ is the ridge regression parameters obtained as the ratio of the residual and marker variances and is represented as λ = σ^2^_*e*_/σ^2^_*u*_. The rrBLUP model was used in this study, as it has the capability of dealing with “large p and small n” with penalized regression and high stability with correlated markers (Endelman, 2011). In our study, we used above GS model with the addition of different parameters in the baseline model.

Several statistical models were used for prediction namely (1) univariate-single trait GS (Uni-GS) model, (2) models using SRI as predictors only, (3) GS model with SRI as a phenotypic covariate, and (4) multivariate GS model where primary and secondary traits were fitted for each environment. The SRI collected at heading and grain filling stages were fitted separately in the model to identify the best stage for data collection for GPC and grain yield. The models used for the analysis are

(1.) Uni-GS used to calculate the GEBVs and represented as

y=μ+Z⁢u+e

Where y is an N × 1 vector of BLUEs for GPC and grain yield for each line, μ is the overall mean, Z is an N × M matrix assigning markers to genotypes and u is a 1 × N matrix of normally distributed marker predictor effects as u ∼ *N*(0, Iσ^2^_*u*_) and e is the residual error with e ∼ *N*(0, Iσ^2^_*e*_). This equation was solved to obtain the GEBVs for all the lines, treating markers as independent variables.

(2.) The univariate model fitting SRI as predictors (SRIr). Only SRI information was used for prediction and the model is represented as

y=μ+Xβ+e

Where *X* is the design matrix for the fixed effect components (SRI) and β is the vector of fixed effect coefficients. Other terms are defined previously. This equation was solved using SRI information as independent variables in the model.

(3.) In a covariate GS model, SRI were fitted as fixed effects (GS + SRI). The equation of the model is represented as

y=μ+Xβ+Zu+e

Where X and Z are the design matrix associating the fixed effects (SRI) and random effects (markers), β is the vector of fixed effect coefficient of each SRI, u is a vector of normally distributed random marker effects as u∼ *N*(0, Iσ^2^_*u*_) and e is the residual error with e ∼ *N*(0, Iσ^2^_*e*_). In this case, both markers and SRI information was used as independent variables in the model for obtaining GEBVs for all the lines.

(4.) Lastly, a multi-variate GS model was used containing primary and secondary traits in the model (Multivariate GS). The equation of the model is represented as

$$\left[\begin{array}{c} y_{1}\\ 1\\ 1\\ 1\\ y\,n \end{array}\right]=\left[\begin{array}{cc} X_{1} & 0\\ 1 & 1\\ 1 & 1\\ 1 & 1\\ 0 & X_{n} \end{array}\right]\,\left[\begin{array}{c} \mu_{1}\\ 1\\ 1\\ 1\\ \mu_{n} \end{array}\right]+\left[\begin{array}{cc} Z_{1} & 0\\ 1 & 1\\ 1 & 1\\ 1 & 1\\ 0 & Z_{n} \end{array}\right]\,\left[\begin{array}{c} u_{1}\\ 1\\ 1\\ 1\\ u_{n} \end{array}\right]+\left[\begin{array}{c} {}_{\epsilon_{1}}\\ 1\\ 1\\ 1\\ {}_{\epsilon_{n}} \end{array}\right]$$

Where n is the number of traits (grain yield or GPC, individual SRI or combination of them), y_1 to n_ represents the vector of BLUEs for the primary (GPC and grain yield) and secondary traits (SRI) y, *X* is a design matrix of fixed effects which simplifies to a vector of 1 for each trait representing the mean only as only markers were entered in the model, *Z* represents the random effect design matrix, $\left[\begin{array}{c} \mu_{1}\\ 1\\ 1\\ 1\\ \mu_{n} \end{array}\right]$ represents the random marker effects, distributed as ∼ *N*(0, G⊗ H) where G is the genomic relationship matrix and H is the variance-covariance matrix, and ∈_1…*n*_ represents the standard normal error, distributed as ∼ *N*(0, I ⊗ R), where I is identify matrix, and R is the residual variance covariance matrix. The covariance matrix H and R were assumed unstructured for the variance estimation. In this multivariate equation, markers are used as independent variables while SRI and primary traits (grain yield or GPC) are used as dependent variables for predicting the GEBVs for all the lines.

### Cross-Validation and Model Performance

The GS models were developed separately for each environment using a subset of the population as the training set to estimate each marker effect. After assessing the marker effects, GEBVs were calculated for the whole population. GS model accuracy is defined as a correlation between GEBVs of predicted individuals and actual phenotypes. GS was performed with five-fold cross-validation by including 80% of the lines in the training model and predicting the GEBVs values of the remaining 20% of the lines under each environment. One replicate consisted of five model iterations where the population was split into five groups, and the testing set was rotated between each group. For accuracy assessment, two 50 replication sets were performed.

Independent validations were performed by training the GS model using the 2014 environment and predicting the GEBVs for remaining two environments. Similarly, the model trained on the 2015 environment was used for predicting GEBVs for the 2016 environment. These validations represent the scenario to predict the performance of a line before planting them in the field for further observations. Furthermore, SRI data were also included in the independent validation GS models. These GS models represent a scenario in the breeding program where SRI have been collected, and the plants are not harvested; therefore, lines will be selected primarily on predicted values.

## Results

### Phenotypic Data Summary

Average grain yield ranged from 1.7 to 2.4 t/ha across the three environments with 2016 having the highest and 2015 being the lowest yielding. Average GPC ranged from 12.2 to 14.4% with 2014 being the highest, and 2015 being the lowest (Supplementary Table 1). Broad-sense heritability of GPC and grain yield were obtained (Table 2). Grain yield and GPC both showed the highest heritability in the 2016 environment. The heritability for grain yield was less than all secondary traits under all environments. The majority of SRI traits have higher heritability than GPC (Tables 2, 3). Within each SRI, 2015 has the lowest heritability (Table 3).

**TABLE 2:** Genotypic variance and heritability for grain protein content and grain yield for a nested association mapping population of spring wheat planted for three environments (2014–2016) in the United States Pacific Northwest.

	Genotypic variance		Heritability^a^	
		
Environment	Grain yield	GPC	Grain yield	GPC
2014	12.62	0.67	0.24	0.62
2015	18.28	0.48	0.42	0.36
2016	14.48	0.99	0.50	0.68

*^*a*^Broad sense heritability.*

**TABLE 3:** Broad sense heritability of eight spectral reflectance indices derived for a spring wheat population planted for three environments (2014–2016) in the United States Pacific Northwest.

Environment	NDVI^a^	NWI^b^	WI^c^	SR^d^	GNDVI^e^	PRI^f^	NCPI^g^	ARI^h^
2014	0.75	0.60	0.71	0.81	0.69	0.93	0.64	0.74
2015	0.66	0.60	0.47	0.57	0.64	0.81	0.55	0.56
2016	0.80	0.74	0.93	0.70	0.72	0.60	0.82	0.76

*^*a*^NDVI, normalized difference vegetation index. ^*b*^NWI, normalized water index. ^*c*^WI, water index. ^*d*^SR, simple ratio. ^*e*^GNDVI, green normalized difference vegetation index. ^*f*^PRI, photochemical reflectance index. ^*g*^NCPI, normalized chlorophyll pigment ratio index. ^*h*^ARI, anthocyanin reflectance index.*

In contrast to heritability, correlation between primary traits (grain yield and GPC) and secondary traits varied significantly across growth stages and environments (Tables 4, 5). This allowed using these correlated responses for predicting the primary traits. Phenotypic correlation of grain yield was higher with SRI at the grain filling stage (Table 4 and Supplementary Table 2), whereas GPC has higher correlation with SRI at the heading stage (Table 5 and Supplementary Table 3). These results were consistent with previous studies where SRI were correlated with grain yield and GPC (Sun et al., 2019). Correlation of grain yield was not significant with most of the SRI under the 2015 environment at the heading and grain filling stages. Genetic correlation of grain yield and GPC with SRI is provided (Supplementary Table 4).

**TABLE 4:** Phenotypic correlation between grain yield and eight spectral reflectance indices derived at the grain filling stage of a spring wheat population planted for three environments (2014–2016) in the United States Pacific Northwest.

Yield	NDVI^a^	NWI^b^	WI^c^	SR^d^	GNDVI^e^	PRI^f^	NCPI^g^	ARI^h^
2014	0.33***	0.36***	0.36***	0.30***	0.37***	0.32***	−0.30***	−0.16***
2015	0.06	0.09*	0.09*	0.03	0.04	0.03	−0.12*	−0.09*
2016	0.20***	0.19***	0.19***	0.19***	0.20***	0.15***	−0.18***	−0.23***

^*a*^NDVI, normalized difference vegetation index. ^*b*^NWI, normalized water index. ^*c*^WI, water index. ^*d*^SR, simple ratio. ^*e*^GNDVI, green normalized difference vegetation index. ^*f*^PRI, photochemical reflectance index. ^*g*^NCPI, normalized chlorophyll pigment ratio index. ^*h*^ARI, anthocyanin reflectance index. ***significant at *P* < 0.0001; *significant at *P* < 0.05.

**TABLE 5:** Phenotypic correlation between grain protein content and eight spectral reflectance indices derived at the heading stage of a spring wheat population planted in three environments (2014–2016) in the United States Pacific Northwest.

GPC	NDVI^a^	NWI^b^	WI^c^	SR^d^	GNDVI^e^	PRI^f^	NCPI^g^	ARI^h^
2014	0.35***	0.35***	0.35***	0.38***	0.34***	0.19***	−0.38***	−0.12*
2015	0.19***	0.21***	0.21***	0.22***	0.28***	0.11*	−0.19***	0.27***
2016	−0.20***	−0.16***	−0.16***	−0.17***	−0.20***	0.02	0.08	0.12*

^*a*^NDVI, normalized difference vegetation index. ^*b*^NWI, normalized water index. ^*c*^WI, water index. ^*d*^SR, simple ratio. ^*e*^GNDVI, green normalized difference vegetation index. ^*f*^PRI, photochemical reflectance index. ^*g*^NCPI, normalized chlorophyll pigment ratio index. ^*h*^ARI, anthocyanin reflectance index. ***significant at *P* < 0.0001; *significant at *P* < 0.05.

### Genomic Selection Within Environments Using All Four Models

For the three environments, GS prediction ability ranged between 0.07 and 0.58 for grain yield using four different models (Table 6). There was an improvement of GS prediction accuracy with inclusion of all SRI traits in the covariate and multivariate GS models. The highest improvement in prediction accuracy was observed for 2014 (35%), followed by 2016 (22%), and negligible effect during 2015 (2.5%). Spectral information collected at the grain filling stage resulted in greater but non-significant improvement in prediction accuracy compared to that of the heading stage. Overall, there was an improvement of 20% prediction accuracy for grain yield by including secondary traits. The multivariate GS model performs as well as the covariate model, with a non-significant difference between the two models. We observed that the highest prediction with the univariate GS model was for the 2016 environment (0.45) compared to the 2014 (0.43), and 2015 (0.40) environments (Table 6). The highest accuracy observed in 2016 was due to the highest broad sense heritability for grain yield for the 2016 environment. However, with the inclusion of secondary traits in the multivariate models, prediction accuracy was highest in the 2014 environment (0.58), which can be attributed to more correlation observed between the grain yield and spectral information (Table 6). In this way, we observed that models perform differently across the environment’s and their performance depends upon the trait heritability and correlation with secondary traits.

**TABLE 6:** Genomic selection accuracies for three different environments (2014–2016) using univariate GS model, all spectral reflectance indices in a univariate model at heading and grain filling stage (SRIr), GS + SRI in a covariate model with SRI as covariate, and multivariate GS model for prediction of grain yield and grain protein content in a spring wheat NAM panel.

Trait			2014	2015	2016

	Model	Stage			
	UniGS		0.43 (0.007)	0.40 (0.007)	0.45 (0.007)
	SRI	Heading	0.44 (0.007)	0.07 (0.009)	0.21 (0.007)
Grain yield		Grain filling	0.50 (0.007)	0.13 (0.009)	0.25 (0.007)
	GS + SRI	Heading	0.52 (0.006)	0.37 (0.007)	0.52 (0.006)
		Grain filling	0.57 (0.007)	0.40 (0.007)	0.55 (0.006)
	Multi-GS	Heading	0.55 (0.006)	0.39 (0.007)	0.53 (0.006)
		Grain filling	0.58 (0.007)	0.41 (0.007)	0.55 (0.006)
	UniGS		0.51 (0.002)	0.55 (0.002)	0.53 (0.002)
	SRI	Heading	0.39 (0.007)	0.37 (0.007)	0.28 (0.009)
GPC		Grain filling	0.31 (0.007)	0.34 (0.009)	0.27 (0.009)
	GS + SRI	Heading	0.63 (0.006)	0.57 (0.006)	0.56 (0.006)
		Grain filling	0.59 (0.005)	0.52 (0.006)	0.55 (0.006)
	Multi-GS	Heading	0.64 (0.006)	0.58 (0.007)	0.56 (0.006)
		Grain filling	0.60 (0.005)	0.54 (0.006)	0.53 (0.006)

*Parenthesis indicates the standard error.*

GS prediction ability ranged between 0.27 and 0.64 for GPC across the three environments (Table 6). Inclusion of all spectral data with genetic markers consistently provided higher prediction accuracies than univariate and SRI information alone. On average, there was an increase in 12% prediction accuracy with multivariate and covariate GS models when all SRI data was used in the models. Similar to the improvement in prediction accuracy for grain yield, there was the highest improvement in 2014 (35%), followed by 2016 (6%) and least in 2015 (3.6%) for GPC. Spectral information collected at the heading stage resulted in a significant (*p* < 0.05) improvement compared to the grain filling stage. The maximum prediction accuracy for GPC was observed during the 2014 environment (0.64), followed by the 2015 (0.58) and 2016 (0.56) environments using multivariate GS models (Table 6). While for the univariate GS model, the maximum prediction accuracy was 0.55 for the 2015 environment (Table 6). This within environment difference in prediction accuracy for GPC can be attributed to environmental variation, which creates this bias. Similarly, to grain yield, the model’s performance was best with the inclusion of secondary traits for 2014 due to more correlation and high heritability of secondary traits for this environment.

### Genomic Selection With Single SRI as Predictor

Along with fitting all the SRI together for predicting GPC and grain yield, individual SRI were used in multivariate GS models. SRI at the heading stage were used to predict GPC, whereas SRI data from the grain filling stage were used for predicting grain yield, as these timings had the highest correlations between traits (Table 6).

**FIGURE 1:**
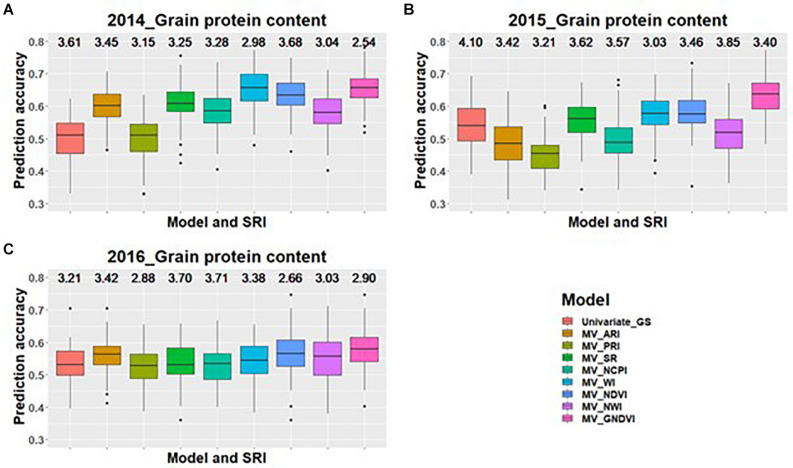
Box plots of prediction accuracies for predicting GPC across three environments (2014–2016) using individual SRI collected at heading, as a predictor in multivariate GS models while results for a univariate GS model is also provided. The figures **(A–C)** represent the model’s performance under the three environments evaluated in this study. The mean squared error for each model is presented above the boxplots.

**FIGURE 2:**
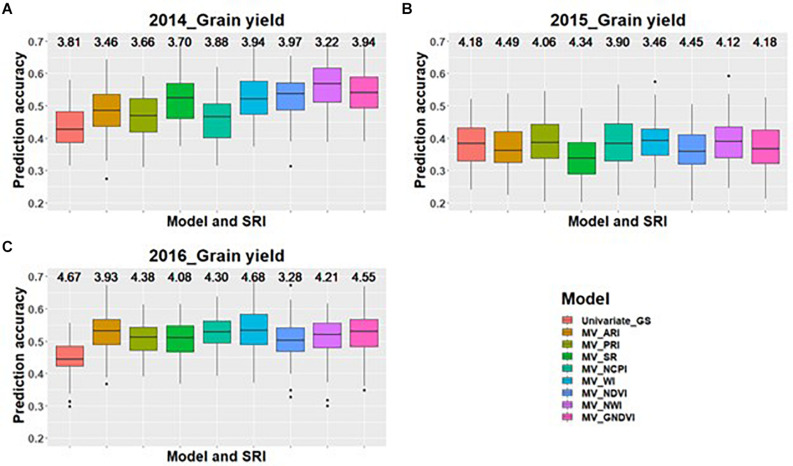
Box plots of prediction accuracies for predicting grain yield across three environments (2014–2016) using individual SRI collected at grain-filling, as a predictor in multivariate GS models while results for a univariate GS model is also provided. The figures **(A–C)** represent the model’s performance under the three environments evaluated in this study. The mean squared error for each model is presented above the boxplots.

Across all environments for predicting GPC, GS models including GNDVI in the multivariate model had the greatest improvement in prediction accuracy for 2014 and 2015 (Figures 1A,B). In 2016, GNDVI and NDVI gave similar increases in prediction accuracy in multivariate GS models (Figure 1C). This suggested that GNDVI is the most important SRI to be included in GS models for predicting GPC. In the case of predictions for grain yield, NWI results in the highest improvement in prediction accuracy for 2014 and 2015 (Figures 2A,B), whereas for 2016 ARI, NCPI, and WI perform better for predicting grain yield (Figure 2C).

### Genomic Selection Across the Environments

**FIGURE 3:**
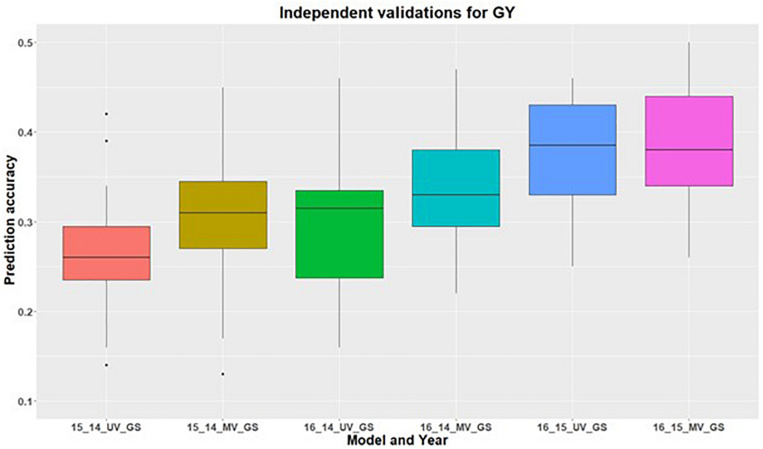
Independent validations using univariate (UV) and multivariate (MV) GS models for predicting grain yield in a United States Pacific Northwest spring wheat mapping population. First number on the *x*-axis represents prediction year, second number represents year for training population and third letter represent the type of GS model used.

**FIGURE 4:**
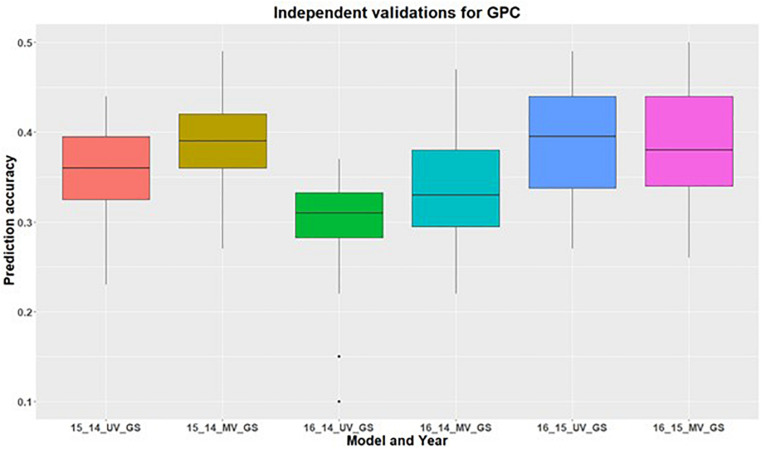
Independent validations using univariate (UV) and multivariate (MV) GS models for predicting grain protein content in a United States Pacific Northwest spring wheat mapping population. First number on the *x*-axis represents prediction year, second number represents year for training population and third letter represent the type of GS model used.

In addition to within environment predictions for both traits, we also assessed the across environment prediction by including spectral information in the models. GS models were trained on previous years data and predictions were made for upcoming years for grain yield (Figure 3) and GPC (Figure 4). Prediction accuracies for independent validations varied across the environments, because of different environmental effects. There was improvement in prediction accuracy for each of the independent validations with inclusion of spectral information in the multivariate GS models, demonstrating the great potential for HTP in wheat breeding and GS. There was higher independent validation for grain yield when GS models were trained on the 2015 environment and predictions were made on the 2016 environment (Figure 3). This was probably a result of the higher phenotypic correlation between SRI and grain yield during this field trial (Figure 3 and Supplementary Table 5). Similar results were obtained for predicting the 2016 environment for GPC using the 2015 environment as the training set (Figure 4 and Supplementary Table 5).

## Discussion

### Prediction Ability With a Univariate, Covariate, and Multivariate GS Models

We evaluated several different models for predicting GPC and grain yield using univariate GS models, SRI in multiple regression, covariate GS models with SRI as a covariate, and multivariate GS models. GS is used in plant breeding to increase genetic gain by reducing the time required to complete the breeding cycle with HTP information improving the accuracy of phenotypic selection (Cobb et al., 2013; Araus and Cairns, 2014). This suggested that combining GS and HTP information could translate into higher genetic gain by reducing breeding cycle time and improving the model’s accuracy. Both Crain et al. (2018) and Sun et al. (2019) combined GS with HTP information for grain yield in wheat and observed improvement in GS accuracy using NDVI and canopy temperature as secondary traits. Herein, we combined eight different SRI collected at heading and grain filling stages for assessing GS performances for GPC and grain yield.

We observed that inclusion of secondary correlated traits into covariate and multivariate GS models resulted in a significant (*p* < 0.05) improvement in prediction accuracies for both traits, which can be attributed to the genetic correlation between primary and secondary traits used in this study. These results are strengthened by GS studies in animals and other crop plants, where improvements in predictions were observed with the inclusion of secondary correlated traits (Tsuruta et al., 2011; Okeke et al., 2017). Another reason for the improvement of predictions in multivariate GS models is attributed to the high heritability of secondary correlated traits when the primary traits have lower heritability, which was the case here for grain yield (Hayashi and Iwata, 2013).

Furthermore, we did not observe any difference in performances of covariate and multivariate GS models for both traits. These observations are consistent with the work of Crain et al. (2018), where they also observed no differences between the performance of covariate and multivariate models for predicting grain yield in wheat with the inclusion of canopy temperature in the multivariate models. Additionally, some studies have shown the superiority of multivariate GS models compared to covariate GS models (Colombani et al., 2012; Rutkoski et al., 2016). Our results thus suggested that secondary correlated information can be used in any of these models for improving the prediction of grain yield and GPC in wheat.

### Genomic Selection Within the Environment

We observed higher and consistent prediction accuracy for predicting both traits under each environmental condition using cross-validation approaches in the univariate GS model. Prediction accuracies are usually higher within the environment than across environment predictions because using a common environment for training and testing the model introduces little bias in assessing the model’s performances. The consistent performance of GS models for all environments could be because univariate GS models can accurately estimate the additive genetic variance component for training the model and requires no environmental components to be included. Furthermore, for within environment predictions, both training and testing populations have the same environmental variations allowing biasness during model training and testing.

Babar et al. (2006) showed that the combined use of SRI collected at booting, heading, and grain filling contributes to the indirect selection response for grain yield in wheat. We observed that SRI collected during grain filling resulted in higher prediction accuracies for grain yield when included in the GS models, because of higher and significant correlation between SRI and grain yield during grain filling compared to at the heading stage. Higher correlation with grain yield could be because grain filling is the main stage for accumulation of carbohydrates in the grain. The difference in grain yield of different wheat lines can be more accurately measured at grain filling compared to heading because of continuous photosynthesis and nutrient translocation happening after heading. This results in higher correlation between SRI and grain yield collected during grain filling compared to at the heading stage.

Grain protein content was accurately predicted at the heading stage, with a higher correlation between SRI and GPC at heading compared to the grain filling stage. The inverse relationship between GPC and grain yield (Avivi, 1978), coupled with the biological mechanics underlying these traits, lends support to these results. The heading stage is important for GPC because, up to this stage, plants accumulate required nitrogen which needs to be translocated to the different plant organs. As senescence starts, and these accumulated products are translocated to different plant organs. This suggests that measuring the nitrogen content is more appropriate at heading, which is directly linked the GPC. SRI collected later at grain filling have less correlation with GPC; this could be because of the saturation of indices.

The inclusion of secondary correlated traits has resulted in improvement in prediction accuracies for grain yield and GPC. This can be attributed to high heritability and genetic correlation between primary and secondary traits. The improved prediction accuracies aid in predicting quantitative traits earlier in the breeding cycle or when seed is limited for performing yield and multi environment trials. Secondary correlated traits also allow the opportunity for modeling the genotype by environmental interaction, ultimately reducing the generations required for variety evaluations. Increased prediction for these quantitative traits aids more accurately predicting such quantitative traits with limited seed availability, which helps in the selection of parents for new crosses earlier in the breeding pipeline. These results suggest that the inclusion of secondary traits have the potential for increasing genetic gain per unit with the inclusion of them in the GS models.

### Genomic Selection With Individual SRI in Multivariate GS Models

When using SRI for predicting GPC and grain yield, we observed that inclusion of individual SRI resulted in significant (*p* < 0.05) improvement for predicting GPC during 2014 and 2015 environment compared to multiple SRI in the model. GNDVI was the best SRI which resulted in the highest increase in GPC prediction accuracy for all three environments, suggesting that there is some strong relationship between GNDVI and GPC. GNDVI measures reflection in the near infra-red and green region of the electromagnetic spectrum (Gitelson et al., 1996). This index provides information about the chlorophyll A concentration in the plants, and it could be possible that this green photosynthetic reflection region is a determinant of GPC in wheat. GNDVI can be used as an indirect selection for GPC because of its significant genetic and phenotypic correlation with GPC and higher heritability than GPC.

Nitrogen is mobilized inside the grain from the soil, and results in the increase of GPC in the wheat grain. Furthermore, early senescence in wheat results in the higher accumulation of GPC and some micronutrients as it increases remobilization of nutrients from the senescing organ to the grain (Olmos et al., 2003; Uauy et al., 2006). GNDVI provides information about nitrogen status in the plant and is linked to the measurement of the reflection for the nitrogen translocation in the plant, which probably results in the better prediction accuracy compared to other indices in this study. The leaf senescence and color changes during the transition of various stages in crop plants are a clear indicator for the accumulation of nutrients and decomposition of pigments which are easily measured by these SRI (Gitelson et al., 1996). NDVI just focuses on the vegetative greenness and plant health, but GNDVI is known to be five times more sensitive to this reflection in the green region of the electromagnetic spectrum (Gitelson et al., 1996). The increased performance in GS models with GNDVI provides insight that plant breeders need to target specific indices for each trait for making predictions.

When predictions were made for grain yield with single and multiple SRI in the GS model, NWI performed best for predicting grain yield under two of the three environments. NWI directly measures the plant hydration status by measuring reflection in the near infrared region (Prasad et al., 2007). The increase in prediction accuracy for grain yield with inclusion of NWI suggests that canopy water status plays an important role for determining grain yield under the rainfed conditions. NWI showed higher genetic correlation compared to the phenotypic correlation observed under each environment. This showed that NWI has an association at the genetic level to grain yield and acts as a powerful tool for determining grain yield. Therefore, higher heritability of NWI than grain yield, larger genetic correlation, and increases in prediction accuracy for grain yield provides ample scope for inclusion of NWI as an indirect estimate for selecting high yielding lines.

We observed that the inclusion of individual SRI is as good, or superior, compared to the addition of multiple SRI in the GS model for GPC. Inclusion of more secondary correlated traits results in multicollinearity issues and made it harder to understand the significance of individual SRI. The multicollinearity arises between the SRI as they are derived from the same region of reflection, either visible or near infrared. This can be avoided by identifying SRI which are highly correlated to each other or provide the same physiological information about the plant traits, and not including them into the multi-variate models. Furthermore, dimension reduction techniques such as partial least square or principal component analysis can be used to extract the latent variables from these SRI which explains the maximum variation between the response and predictors (Wold et al., 2001; Crain et al., 2018). Montesinos-López et al. (2017) showed that with the use of functional splines and functional Fourier models, 250 reflection bands could be used to make predictions for grain yield in wheat while avoiding multicollinearity issues.

Another advantage of including individual SRI compared to multiple SRI, is computational time and convergence problems in the mixed model outcomes. There is no advantage of including a large number of SRI in the GS models if they do not increase the prediction accuracy. Schulthess et al. (2016) also showed that inclusion of multiple traits in GS models did not result in improved predictions compared to inclusion of the single best correlated features for predicting grain yield in rye (*Secale cereale*), validating our results for inclusion of the single most influential SRI in the GS models. In this context, due to issues associated with inclusion of large number of SRI in the GS models, it is advisable to select the best SRI for making predictions. In this study, we concluded that GNDVI and NWI result in the highest improvement in the prediction accuracy for GPC and grain yield, respectively, and should be used in selecting improved lines. Furthermore, these indices have higher genetic correlation and heritability for inclusion in the GS models and will ultimately translate to increase the genetic gain in wheat breeding.

### Genomic Selection Across Environments Without Inclusion of Secondary Traits

We also applied across cycle predictions providing realistic scenarios for the use of GS in plant breeding programs by testing the performance of lines in untested environments. There was a significant (*p* < 0.05) decrease in across environment prediction accuracy compared to cross-validation prediction accuracy. This is due to the different environmental conditions in training and testing environments compared to the common environment for the cross-validation predictions. Furthermore, univariate GS models are not able to model the genotype by environment interactions and provide an opportunity for inclusion of secondary correlated traits or genotype by environment interactions into the GS models to explain such variations. The other reason for the decrease in prediction accuracy is that both these traits are polygenic. Hence, training of a population in one environment results in the prediction of marker effect under that environment only, completely ignoring non-genetic variations (González-Recio et al., 2014). When that same model was used for predicting phenotypes in unknown environments, it results in less accuracy because the real phenotype not only depends upon the genetic effect, but also on environment and genotype by environmental interactions.

The highest independent prediction accuracy for grain yield was observed for the 2016 environment when the GS model was trained on the 2015 environment. This is because the genotypic variance explained for grain yield was highest for the 2015 environment compared to the other two environments (Table 2). Higher genotypic variance for the 2015 environment results in the accurate training of the GS models which explain more genetic variation, and ultimately results in better predictions of grain yield for 2016. Another reason for this high prediction for these two environment combinations could be because of the larger phenotypic correlation of grain yield for these two environments (Supplementary Table 5). There was variation in the correlation between traits across environments because of the varying environment and genotype by environment interactions, providing the importance of conducting multi environmental and replicated trails.

### Improvement of Across Environments Prediction Accuracy With the Inclusion of Secondary Traits

Previous studies by Rutkoski et al. (2016) and Crain et al. (2018) have demonstrated improvement in prediction accuracies for grain yield using secondary traits (NDVI and canopy temperature) within and across cycles. Rutkoski et al. (2016) observed a maximum improvement of 70% prediction accuracy for grain yield with secondary traits in multivariate models. Such high accuracy in their study was attributed to many replications, large training population, frequent collection of spectral information across all replicates, and correctness for days to headings in the models. However, Crain et al. (2018) concluded that prediction accuracy in multivariate models varies from −33 to 7% for predicting grain yield with the inclusion of secondary traits. Herein, we included secondary traits from the tested environments for making predictions and observed the improvement in prediction accuracies for both traits, which vary from 1–10%. This improvement in prediction accuracy suggests that secondary traits can accommodate some amount of environmental effect, and hence improve the model performance. The other reason for the improvement in prediction accuracy is the genetic correlation between primary and secondary traits and higher heritability of the secondary traits. Improvement in prediction accuracy across environments provides an opportunity for inclusion of the best SRI in the GS model for each trait. These collected SRI can aid in understanding the genotype by environment predictions which might be present under the testing environment.

We did not observe any improvement in independent prediction accuracies for GPC in 2016, which can be attributed to the negative correlation observed between GPC and SRI in 2016 compared to positive correlation in other environments. This suggests that GS selection models used for across cycle predictions are problematic if the secondary correlated traits are affected by environmental conditions and do not account for unpredictable genotype by environment interactions. These findings provide evidence that increased prediction in the untested environment is governed by the secondary correlated traits.

The approach used for across cycles predictions in this study resembles a breeding program where SRI has been collected, and plots are not yet harvested to get information for grain yield and GPC. This allows the breeders to make the selection based on these predicted GEBVs by incorporation of secondary traits in the multi-trait GS models. Previously, it has been shown that these secondary correlated traits have the potential for indirect selection in the PNW for spring and winter wheat breeding (Gizaw et al., 2018a,b). This study demonstrated the potential of inclusion of secondary traits for predicting GPC and grain yield for spring wheat within and across cycles. These increases in GS selection accuracy will aid in increasing the genetic gain per unit time and cost. The findings from this study can be applied in other crops with selection of appropriate secondary traits and identification of appropriate growth stages for collecting them.

## Conclusion

Our study demonstrates the improvement in GS prediction accuracies for grain yield and GPC in wheat with the inclusion of secondary correlated traits in the models and identifies the most effective SRI and plant growth stage for secondary data collection. We observed a vital role of secondary traits for improving the predictions for both within and across cycle predictions. On average, there was an improvement in prediction accuracies of 20% for grain yield and 12% for GPC. Moreover, we observed that secondary traits have the potential to improve independent validations, showing their capabilities to accommodate for different environmental effects in the models. This study shows the potential of combining genomics and HTP for improving selection in wheat breeding programs and can be transferable to other plant breeding programs. Inclusion of HTP and GS in a plant breeding program will ultimately improve the genetic gain by increasing the selection accuracy and reducing the cycle time.

## Data Availability Statement

The raw data supporting the conclusion of this article will be made available by the authors, without undue reservation.

## Author Contributions

KS analyzed the data, conceptualized the idea, and drafted the manuscript. PM assisted in genotyping curation and edited the manuscript. ML collected the phenotypic data. MP edited the manuscript, conducted field trials, and obtained the funding for the project. AC edited the manuscript, conducted field trials, and obtained the funding for the project. All authors read and approved the final manuscript.

## Conflict of Interest

The authors declare that the research was conducted in the absence of any commercial or financial relationships that could be construed as a potential conflict of interest.
