# Supplementary material for: Combining Genomic and Phenomic Information for Predicting Grain Protein Content and Grain Yield in Spring Wheat
Source: Front Plant Sci. 2021 Feb 12;12:613300. doi: 10.3389/fpls.2021.613300 (PMC7907601; doi:10.3389/fpls.2021.613300)
Supplement: Supplementary file 1 [file Data_Sheet_1.docx]

**Supplementary Table 1.** Average grain yield and grain protein content for the nested association mapping population of spring wheat planted for three years (2014-16) in the US Pacific Northwest.

| Environment | Grain yield (t/ha) | GPC (%) |
| --- | --- | --- |
| 2014 | 1.9 | 14.4 |
| 2015 | 1.7 | 12.2 |
| 2016 | 2.4 | 12.6 |

| **Supplementary Table 2.** Phenotypic correlation between grain yield and eight spectral reflectance indices derived in this study at the heading stage of spring wheat population planted for three environments (2014-16) in the US Pacific Northwest. | | | | | | | | |
| --- | --- | --- | --- | --- | --- | --- | --- | --- |
| Yield | NDVI ^a^ | NWI ^b^ | WI ^c^ | SR ^d^ | GNDVI ^e^ | PRI ^f^ | NCPI ^g^ | ARI ^h^ |
| 2014 | 0.20*** | 0.19*** | 0.19*** | 0.15*** | 0.26*** | 0.21*** | -0.13* | -0.16*** |
| 2015 | 0.03 | 0.00 | 0.00 | 0.01 | 0.05 | 0.07 | -0.07 | -0.09* |
| 2016 | 0.15*** | 0.05 | 0.05 | 0.16*** | 0.20*** | 0.16*** | -0.15*** | -0.23*** |
| ^a^ NDVI, Normalized difference vegetation index; ^b^ NWI, Normalized water index; ^c^ WI, Water index; ^d^ SR, Simple ratio; ^e^ GNDVI, Green normalized difference vegetation index; ^f^ PRI, Photochemical reflectance index; ^g^ NCPI, Normalized chlorophyll pigment ratio index; ^h^ ARI, Anthocyanin reflectance index; *** significant at P < 0.0001; ** significant at P < 0.001; * significant at P < 0.05 | | | | | | | | |

| **Supplementary Table 3.** Phenotypic correlation between grain protein content and eight spectral reflectance indices derived in this study at the grain filling stage of spring wheat population planted for three environments (2014-16) in the US Pacific Northwest. | | | | | | | | |
| --- | --- | --- | --- | --- | --- | --- | --- | --- |
| GPC | NDVI ^a^ | NWI ^b^ | WI ^c^ | SR ^d^ | GNDVI ^e^ | PRI ^f^ | NCPI ^g^ | ARI ^h^ |
| 2014 | 0.26*** | 0.27*** | 0.27*** | 0.28*** | 0.29*** | 0.10 | -0.20*** | -0.12* |
| 2015 | .08* | -0.03 | -0.02 | 0.11* | 0.18*** | 0.01 | 0.02 | 0.27*** |
| 2016 | -0.19*** | -0.22*** | -0.22*** | -0.13*** | -0.15*** | -0.10* | 0.13* | 0.12* |
| ^a^ NDVI, Normalized difference vegetation index; ^b^ NWI, Normalized water index; ^c^ WI, Water index; ^d^ SR, Simple ratio; ^e^ GNDVI, Green normalized difference vegetation index; ^f^ PRI, Photochemical reflectance index; ^g^ NCPI, Normalized chlorophyll pigment ratio index; ^h^ ARI, Anthocyanin reflectance index; *** significant at P < 0.0001; ** significant at P < 0.001; * significant at P < 0.05 | | | | | | | | |

**Supplementary Table 4.** Genetic correlation of spectral reflectance indices with grain yield and grain protein content across three years.

| Trait | NDVI ^a^ | NWI ^b^ | WI ^c^ | SR ^d^ | GNDVI ^e^ | PRI ^f^ | NCPI ^g^ | ARI ^h^ |
| --- | --- | --- | --- | --- | --- | --- | --- | --- |
| Grain yield | 0.73 | 0.65 | 0.59 | 0.68 | 0.65 | 0.52 | 0.56 | 0.59 |
| GPC | 0.61 | 0.65 | 0.69 | 0.72 | 0.70 | 0.48 | 0.53 | 0.55 |
| ^a^ NDVI, Normalized difference vegetation index; ^b^ NWI, Normalized water index; ^c^ WI, Water index; ^d^ SR, Simple ratio; ^e^ GNDVI, Green normalized difference vegetation index; ^f^ PRI, Photochemical reflectance index; ^g^ NCPI, Normalized chlorophyll pigment ratio index; ^h^ ARI, Anthocyanin reflectance index; all genetic correlations are significant at p < 0.05 | | | | | | | | |

**Supplementary Table 5.** Phenotypic correlation of grain protein content and grain yield across the environments.

| Environments | Grain yield | Grain protein content |
| --- | --- | --- |
| 2014-15 | 0.47 | 0.49 |
| 2014-16 | 0.42 | 0.42 |
| 2015-16 | 0.55 | 0.59 |
| All correlations were significant at p < 0.05 | | |
